# Secondary Prevention Bundle for Drug-Related Problems in an Integrated Healthcare Setting: A Pragmatic Observational Implementation-Effectiveness Study

**DOI:** 10.3390/pharmacy14060134

**Published:** 2026-09-15

**Authors:** Jordi Fernández-Morató, Jesús Ruiz-Ramos, Mireia Fuster-Barrera, Alícia Antón-López, Eduardo Ortiz-Del Romero, Adrián Plaza-Ruiz, Cristina Roure-i-Nuez

**Affiliations:** 1 Pharmacy Department, Consorci Sanitari de Terrassa (CST), 08227 Terrassa, Spain; mfuster@cst.cat (M.F.-B.); aanton@cst.cat (A.A.-L.); eortiz@cst.cat (E.O.-D.R.); 2 Pharmacy Department, Hospital de la Santa Creu i Sant Pau, 08026 Barcelona, Spain; jruizr@santpau.cat (J.R.-R.); aplaza@santpau.cat (A.P.-R.)

**Keywords:** drug-related problems, medication reconciliation, emergency department, hospital readmission, integrated healthcare organization, secondary prevention, clinical pharmacy

## Abstract

This study evaluates the implementation and associated 30-day outcomes of the “Medication Code” (MC), a secondary prevention bundle for drug-related problems (DRPs), implemented at the Consorci Sanitari de Terrassa (CST), an integrated healthcare organization in Catalonia. The bundle, which combines medication reconciliation, structured medication review, and coordinated communication with primary care, was previously shown to reduce 30-day readmissions in a randomized trial at a large tertiary hospital. Between May 2024 and December 2025, 185 patients attending the emergency department (ED) at CST for a DRP received the secondary prevention intervention bundle. The study assesses the association between implementation of the recommendations and 30-day outcomes, not the effect of the MC bundle itself. Treatment optimization proposals were implemented in 72.4% of patients, either at discharge or during primary care follow-up, supported by a multiprofessional integrated team of pharmacists and primary care practitioners. Implementation of the pharmacotherapeutic proposals was associated with an absolut risk reduction of 30-day ED revisits and DRP-related readmissions. These findings are consistent with previous observations from the original MC program. However, because all included patients received the intervention bundle and outcomes were compared according to implementation status, the study cannot estimate the effectiveness of the MC bundle itself.

## 1. Introduction

Over recent decades, demographic aging and the epidemiological transition toward chronic multimorbidity have profoundly transformed healthcare systems. The expansion of long-term pharmacotherapy has led to a sustained increase in polypharmacy, particularly among older adults and patients with complex chronic conditions. Therefore, drug-related problems (DRPs) and adverse drug events (ADEs) have emerged as major determinants of patient safety and healthcare utilization worldwide [1,2,3].

DRPs are clinically relevant events that interfere with therapeutic outcomes and may lead to substantial morbidity and mortality. It is estimated that up to 12% of hospital admissions [4], 5% of hospital readmissions [5], and between 10–20% of emergency department (ED) visits are related to DRPs [6]. Nevertheless, 33% of patients presenting with a DRP revisit the ED within 30 days after discharge [7]. Depending on the methodology and setting, between 30% and 80% of medication-related ED visits have been classified as potentially avoidable through optimized prescribing, reconciliation, monitoring, and care coordination strategies [8,9,10]. Despite this high degree of preventability, structured secondary prevention strategies in the ED remain inconsistently implemented, and multicenter registries capturing post-discharge outcomes are scarce.

Multidisciplinary intervention bundles integrating medication reconciliation, structured medication review, patient education, and coordinated communication with primary care have shown promising results. Juanes et al. demonstrated that a secondary prevention bundle for DRPs, known as the “Medication Code” (MC), implemented in patients attending the ED who had already experienced a DRP, reduced 30-day all-cause hospital readmission rates [11]. The MC bundle consisted of optimization of chronic prescribing, improvement of patient understanding and adherence to the therapeutic regimen, and care coordination with primary and intermediate care after hospital discharge. Similarly, pharmacist-led programs in emergency settings have reported improvements in medication safety indicators and reductions in healthcare reutilization [12,13].

Based on these favourable results, the Catalan Health Service identified the MC as one of the 18 transformative programs of the Catalan healthcare system and promoted the analysis of scalability and implementation of the MC DRP prevention program into routine clinical practice in other Catalan hospitals.

Scaling the MC program beyond its original tertiary hospital setting is therefore a strategic priority, but real-world conditions introduce variability that may affect its effectiveness. Our study examines whether full implementation of pharmacotherapeutic recommendations at hospital discharge generated within the MC framework is associated with healthcare utilization outcomes in an integrated healthcare setting.

## 2. Materials and Methods

### 2.1. Study Setting

Consorci Sanitari de Terrassa (CST) is as a territorially based Integrated Healthcare Organization (IHO) that integrates acute hospital care, primary and intermediate care, and transitional care services through shared care pathways and interoperable clinical information systems. This organizational structure enables continuity of medication-related interventions initiated in the ED, facilitates bidirectional communication between hospital and primary care teams, and allows capture of post-discharge outcomes through electronic health records and shared regional registries.

### 2.2. Study Design

We conducted a pragmatic observational implementation-effectiveness study to evaluate the implementation of the MC secondary prevention bundle and its association with DRPs and 30-day hospital usage outcomes under routine clinical practice conditions. The study focused on patients attending the ED for a DRP in whom a pharmaceutical intervention proposal was prospectively generated during routine clinical practice in the index episode. The outcomes were analysed through retrospective review of electronic health records.

Rather than conducting a comparison with an external cohort without a structured intervention, the analysis focused on internal differences according to the degree of intervention implementation. Outcomes were compared between patients in whom the proposed interventions were fully implemented at hospital discharge (as defined in Section 2.8) and those in whom they were partially or not implemented (both considered not implemented). Since every included patient was exposed to the bundle, the estimated associations refer to implementation of its pharmacotherapeutic recommendations, not to the effect of the bundle itself.

This design reflects real-world implementation conditions, where generation of an intervention proposal does not necessarily translate into effective treatment modification, and allows assessment of the clinical outcomes associated with actual implementation rather than mere intervention exposure. Allocation to implementation versus non-implementation was not randomized and was influenced by patient complexity, clinical decision-making at discharge, and follow-up in primary care; therefore, adjusted and propensity score–based analyses were prespecified to address confounding.

The intervention model, structure of proposals, and definition of DRPs followed the bundle tested by Juanes et al. [11], adapted to CST care pathways and information systems.

### 2.3. Definition and Identification of DRPs

A DRP was defined as a health problem experienced by the patient related to the use or non-use of medications, in accordance with previous definitions [11,13].

Potential medication-related presentations were identified during the index ED episode as part of routine clinical practice. Following initial screening, an ED clinical pharmacist performed a structured assessment to determine whether medication use or non-use plausibly contributed to the clinical presentation.

The assessment incorporated review of the patient’s active medication list, direct patient and/or caregiver interview when feasible, actual medication use and adherence, recent medication initiation, discontinuation or dose modification, medication reconciliation findings, relevant laboratory and clinical data, known pharmacological effects, and the temporal relationship between medication exposure and the presenting clinical problem. Information documented by the treating medical team was also considered.

For clinical presentations with multiple potential causes, such as falls, somnolence, electrolyte abnormalities, or exacerbation of chronic disease, the presence of medication exposure alone was not considered sufficient to classify the episode as a DRP. Medication use or non-use had to be considered a clinically plausible contributor to the index presentation after integration of the available clinical, pharmacological, and temporal information. When uncertainty remained, attribution was discussed with the treating physician and/or the multidisciplinary team.

### 2.4. Study Recruitment

Patients were included between 1 May 2024, and 31 December 2025, with 30-day follow-up after hospital discharge (final follow-up completed on 31 January 2026).

### 2.5. Inclusion and Exclusion Criteria

**Figure 1:**
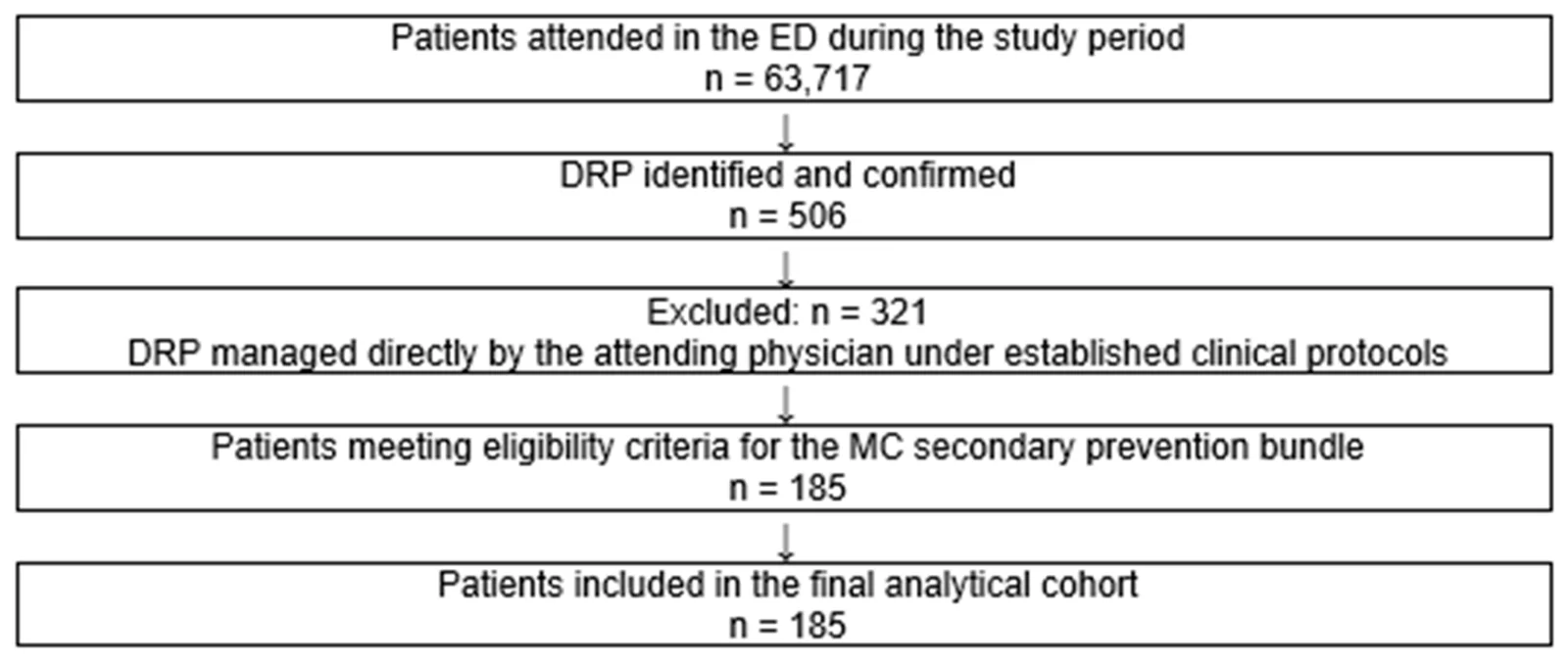
STROBE flow diagram of study population selection DRP: drug-related problem; ED: emergency department.

Potentially eligible patients were identified during routine clinical practice through systematic screening of ED presentations by a clinical pharmacist. The inclusions criteria were adult patients (≥18 years) attending the ED, with or without subsequent hospital admission, who met both of the following criteria: (1) a DRP contributing to the index ED presentation was identified and confirmed; and (2) the clinical pharmacist generated at least one pharmacotherapeutic recommendation intended to reduce the risk of recurrence or optimize medication-related care. DRP secondary to chemotherapy treatment, DRP associated with suicide attempts and DRP occurring in patients in a terminal condition (life expectancy < 30 days) were not eligible for screening and are therefore not included among the 506 confirmed DRP. Patients whose DRP was managed directly by the attending physician under established clinical protocols were also excluded from analysis. Figure 1 illustrates the STROBE flow diagram detailing DRP-patient identification, exclusions, patients meeting eligibility criteria and final analytical cohort.

### 2.6. Variables

Baseline variables were collected from the electronic health record at the index episode and included:

- Demographic data: age, sex;
- Comorbidity burden, measured using the Adjusted Morbidity Group (GMA) [14], which classifies individuals according to the distribution of a multimorbidity index in the general population, into baseline risk (1), low risk (2), moderate risk (3), high risk (4), and very high-risk (5) categories;
- Patient complexity according to the Chronicity Prevention and Care Programme (Health Plan for Catalonia 2011–2015), distinguishing patients with chronic diseases and complex care needs (PCC) and patients with advanced chronic disease and a life-limiting prognosis (MACA) [15];
- Polypharmacy, expressed as the number of chronic medications;
- Diagnosis associated with the DRP;
- The Anatomical Therapeutic Chemical (ATC) group involved in the DRP;

There were no missing data for the variables included in the primary analyses or for the recorded 30-day outcomes; therefore, no statistical imputation was performed.

### 2.7. DRP Prevention Bundle Approach

Patients received a secondary prevention bundle for DRPs, initiated in the ED and delivered once during the index episode by a single ED clinical pharmacist, replicating the structure of the DRP prevention bundle described by Juanes et al. [11]. The bundle was implemented by the ED clinical pharmacist in collaboration with ED physicians and other specialists (geriatricians, internists) and was coordinated with three clinical pharmacists from the primary care network.

The intervention included three domains:

1. Medication reconciliation and best possible medication history. Within CST, medication reconciliation during ED admission is structurally supported by integration with the regional electronic prescribing system (SIRE) [16]. At admission, the ED physician selects the active chronic medications to be continued, which are automatically transferred to the inpatient prescription module, and predefined therapeutic interchange protocols may be applied automatically. The platform also includes a chronic treatment verification module comparing SIRE-registered medication with patient-reported use; in routine practice this verification may be performed by any professional and is not systematically documented. The ED clinical pharmacist performed a structured and documented medication verification process that systematically included:

- Review of active medications listed in SIRE;
- Direct patient and/or caregiver interview to confirm actual use, adherence, recent changes, and discrepancies if necessary;
- Resolution and documentation of discrepancies when clinically relevant;
- Confirmation of therapeutic interchange when applicable.

2. Structured, patient-centered medication review. A structured review was conducted using the three-step multidisciplinary method described in the reference model:

- Patient-centered assessment to define the overall goal of care (survival, functional status, symptom control);
- Diagnosis-centered assessment to evaluate conditions and diagnosis-specific treatments in alignment with the care goals;
- Medication-centered assessment to analyse the benefit–risk balance and appropriateness of each drug in the patient’s clinical context.

3. Structured report to the patient’s primary care team. Proposals were exposed and discussed in the Pharmacy Department team meetings, and a standardized primary care intervention was stablished: a specific protocol for communicating recommendations to the primary care physician, specific MC training, and a scheduled slot in the physician’s agenda for review and, where appropriate, implementation of the recommendations. The report included: (i) identification of the DRP that prompted the ED visit; (ii) discharge treatment; and (iii) recommendations to optimize treatment and medium- and long-term follow-up.

### 2.8. Outcomes and Follow-Up

The primary evaluation horizon was 30 days after discharge of the index ED episode. Outcomes were defined as:

- 30-day ED DRP-related revisit (primary endpoint)

Secondary endpoints:

- 30-day ED any cause revisit
- 30-day unplanned DRP-hospital readmission
- 30-day unplanned any cause hospital readmission

Implementation status was classified at the patient level as (i) full implementation, when all proposals generated during the index ED episode were adopted and documented in the clinical record at discharge, or (ii) non-implementation, when proposals were not fully adopted at hospital discharge. Exposure was therefore dichotomized as fully implemented versus not implemented. The implementation of treatment optimization proposals was assessed both at hospital discharge and during primary care 30 days follow-up.

The causality between ED revisits and DRPs was independently evaluated by the ED pharmacist and confirmed by the attending ED physician, who remained blinded to the implementation status of the proposed pharmacotherapeutic intervention. Outcomes for hospital ED revisits and readmissions and implementation status were ascertained through retrospective reviews of electronic health records and shared regional registries capturing post-discharge healthcare utilization.

### 2.9. Statistical Analysis

Baseline characteristics were summarized using descriptive statistics. The primary analyses assessed the association between implementation status of pharmaceutical interventions and 30-day outcomes. No formal a priori sample size calculation was performed. The sample size was determined by the complete cohort of eligible patients included in the MC implementation program during the predefined study period.

Continuous variables were described as mean and standard deviation and compared using Student’s *t*-test. Categorical variables were expressed as frequencies and percentages and compared using Pearson’s chi-square test or Fisher’s exact test when appropriate. Adjusted associations between implementation status and outcomes were estimated using multivariable logistic regression models. To ensure model stability, avoid overfitting, and address severe multicollinearity, the multivariable logistic regression model was constrained to key prespecified clinical predictors and variables meeting our univariable screening threshold (*p* < 0.10).

To control for baseline confounding in this observational study, a propensity score (PS) analysis was performed including all key baseline covariates: age, sex, GMA complexity tier, number of active chronic medications, and PCC/MACA status. Primary adjustment was conducted using Inverse Probability of Treatment Weighting (IPTW) to estimate the Average Treatment Effect. As a sensitivity analysis, 1:1 nearest-neighbor matching without replacement was executed using a caliper of 0.2 standard deviations of the logit PS. Finally, adjusted outcome analyses were performed using IPTW.

## 3. Results

**Figure 2:**
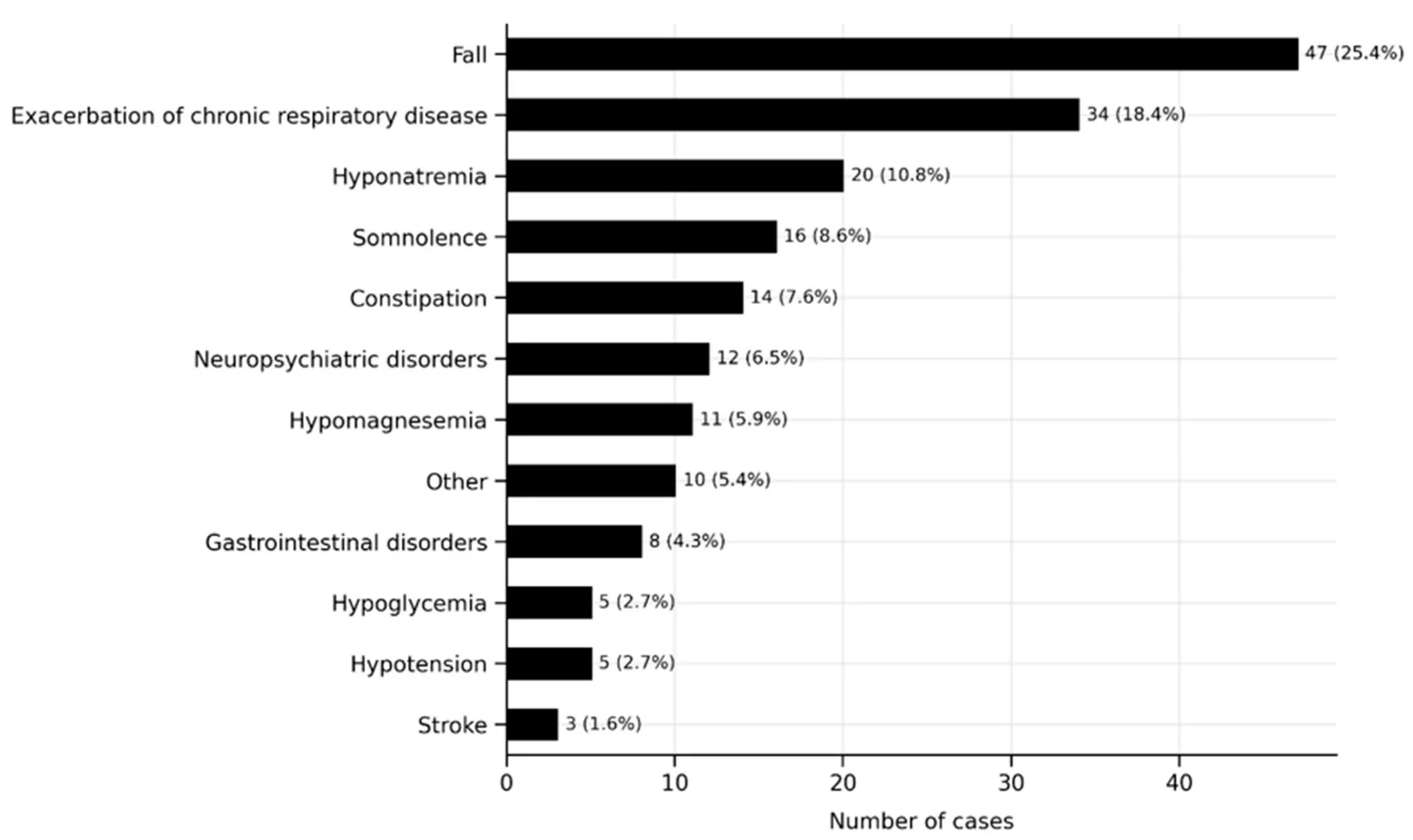
DRPs triggered activation of the secondary prevention bundle during the initial ED visit.

During the study period, a total of 185 patients received the MC secondary prevention bundle. The most common DRPs that motivated the intervention were falls (47; 25.4%), exacerbation of chronic respiratory disease (34; 18.4%), and hyponatremia (20; 10.8%), followed by somnolence (16; 8.6%) and constipation (14; 7.6%). Neuropsychiatric disorders (12; 6.5%) and hypomagnesemia (11; 5.9%) were also frequent (Figure 2).

A total of 337 pharmacotherapeutic interventions were generated (mean 1.82 interventions per patient, median 1, range 1–6). Because individual patients could receive more than one recommendation, all effectiveness analyses were conducted at the patient level according to implementation status.

Among the 185 included patients, 101 (54.6%) achieved full implementation of all medication review recommendations at discharge and were therefore classified as implemented at discharge. The remaining 84 patients (45.4%) were classified as not fully implemented at discharge, including 19 patients with partial implementation and 65 with no implementation.

Recommendations were communicated to primary care in 78 of these 84 patients (92.9%). Within 30 days after discharge, 33 patients (42.3%) achieved full implementation in primary care, with a mean implementation of 7 ± 6 days after discharge. Overall, considering both hospital discharge and subsequent primary care implementation, 134 patients (72.4%) achieved full implementation and 51 patients (27.6%) remained not fully implemented.

Baseline characteristics are reported in Table 1. In the multivariable model, the number of drugs remained a significant independent predictor (OR per additional drug: 0.919, 95% CI 0.857–0.984; *p* = 0.016), indicating that the probability of full implementation decreases as the number of medications increases.

The PS showed adequate common support across groups, ranging from 0.252 to 0.689 in the Not Implemented group and 0.311 to 0.814 in the Implemented group, indicating satisfactory overlap without requiring extreme weight trimming. Following IPTW weighting, the Effective Sample Size (ESS) was ESS = 177.9 patients overall (80.8 in the Not Implemented group and 97.1 in the Implemented group).

As detailed in Supplementary Table S1, baseline differences between patients with and without implementations at hospital discharge were generally modest. The largest imbalances were observed for the number of active medications and GMA complexity tier, while smaller differences were noted for sex, PCC/MACA status and age. Following IPTW adjustment, covariate balance was substantially improved across all measured parameters, with all post-weighting SMD values falling below the conventional 0.10 threshold (range: 0.012–0.045), suggesting that IPTW adequately attenuated observed baseline differences.

Regarding 30-day outcomes (Table 2), patients whose pharmacotherapeutic proposals were implemented at hospital discharge experienced lower rates of DRP-related ED revisits and all-cause ED revisits compared with those without implementation at discharge. Consistent patterns were observed for unplanned hospital readmissions, with lower rates of both DRP-related readmissions and all-cause readmissions among implemented patients. These estimates represent the crude outcome risks observed in the study cohort.

IPTW-adjusted analyses consistently favoured implementation at hospital discharge (Supplementary Table S2). Significant reductions were observed for both all-cause and DRP-related ED revisits, as well as for DRP-related hospital readmissions. The largest effect was observed for DRP-related ED revisits, with an absolute weighted risk reduction of 20.3 percentage points and an odds ratio of 0.06 (95% CI 0.01–0.30). For all-cause hospital readmissions, the estimated effect also favoured implementation, although statistical uncertainty was greater and the confidence interval crossed the null value.

Of the 33 patients whose pharmacotherapeutic proposals were implemented after discharge during primary care follow-up, only two experienced an ED revisit or readmission after implementation.

## 4. Discussion

This implementation study suggests that a structured secondary prevention bundle for drug-related problems was associated with a lower rate of 30-day DRP-related ED revisits, the primary outcome, among patients attended for a DRP. Similar patterns were observed for all-cause ED revisits and hospital readmissions. These findings are consistent with the results reported by Juanes et al. [11] and suggest that successful implementation of pharmacotherapeutic recommendations may be associated with more favourable short-term outcomes in routine clinical practice.

Whereas Juanes et al. [11] conducted a randomized trial in a large tertiary hospital, the present work provides complementary evidence under real-world conditions in a territorially based integrated healthcare organization. This suggests that the outcomes associated with the MC may be attainable outside an experimental setting and may persist when the intervention is embedded in routine care pathways, supporting transferability of the original model—although the observational design does not permit an estimate of the effect of the bundle itself.

A distinguishing feature of our approach is that the analysis focused on effective implementation of pharmacotherapeutic proposals rather than on exposure alone, highlighting the importance of their adoption and persistence across care transitions. This may partly explain the variability in effects reported in other medication review programs [17].

The study population was characterized by a high level of clinical and pharmacotherapeutic complexity, with substantial polypharmacy and a considerable proportion of complex chronic patients or patients with advanced chronic disease. This profile is consistent with the literature, which identifies these groups as being at greatest risk of drug-related problems and repeated healthcare utilization [5,18]. In this context, the finding that the number of medications was independently associated with a lower likelihood of full implementation of recommendations suggests the presence of additional barriers in patients with extreme polypharmacy, potentially related to clinical complexity, therapeutic inertia, or organizational challenges specific to this population [19]. This result underscores the need to strengthen decision-support strategies and care coordination mechanisms for these high-risk profiles.

From a methodological standpoint, the combined use of multivariable regression models and propensity score–based analyses enhance the credibility of the findings, in line with what was previously emphasized in other studies regarding the importance of adequately adjusting for baseline patient complexity [11,12,13]. The consistency in the direction of estimates across different analytical approaches provides some reassurance regarding their stability with respect to measured covariates. Nevertheless, residual and unmeasured confounding remain possible.

Finally, our study highlights the role of the CST integrated care model as a key facilitator of the intervention effect. Structured communication with primary care, often mediated by the primary care pharmacist, enabled the implementation of a substantial proportion of recommendations that had not been adopted at hospital discharge. This finding reinforces the notion that secondary prevention programs for DRPs should not be conceived as isolated, episode-based interventions limited to the ED, but rather as transversal processes that rely on continuity of care to achieve their full potential [20].

This study should be interpreted considering several limitations. Its observational design prevents causal inference; implementation of pharmacist recommendations was not randomized and unblinded during follow up. Because the sample size was determined by the available implementation cohort rather than by an a priori power calculation, the study may have had limited statistical power for outcomes with a small number of events, particularly hospital readmission and DRP-related endpoints. Despite propensity score adjustment, residual confounding and implementation-related selection bias cannot be excluded. Factors influencing the feasibility and acceptance of recommendations, such as patient clinical complexity, organizational context, and unmeasured clinical characteristics, may have affected both implementation status and subsequent healthcare utilization. Therefore, the observed associations should not be interpreted as causal and warrant confirmation in prospective controlled studies.

The single-center nature of the study within an integrated healthcare organization may also limit generalizability to settings without shared electronic records or established primary care pharmacy pathways. We also cannot exclude admissions occurring in other hospitals outside our integrated network, as follow-up relied on institutional electronic records and shared registries not always available. Finally, implementation was based on documentation in clinical records, which may have resulted in misclassification if some recommendations were adopted but not fully recorded.

Despite these limitations, the study has several strengths, including its pragmatic design, the inclusion of real-world ED patients with high clinical and pharmacotherapeutic complexity, the assessment of implementation fidelity rather than intervention exposure alone, and the evaluation of outcomes across care transitions within an integrated healthcare organization and a cross-setting pharmacist team.

## 5. Conclusions

Full implementation of pharmacotherapeutic proposals included in the MC secondary prevention bundle was associated with lower healthcare utilization during the 30 days following discharge, particularly for drug-related revisits. However, because of the observational design, potential residual confounding, and the absence of a usual-care comparator, these findings should be interpreted as associative and hypothesis-generating rather than evidence of effectiveness. Future prospective controlled studies are needed to confirm these findings and to evaluate the effectiveness of this intervention in routine clinical practice, as well as to further define its potential value within integrated healthcare organizations.

## Figures and Tables

**Table 1:** Baseline characteristics and univariate analysis.

Variable	Category	No Implementation (n = 84)	Implementation (n = 101)	*p*-Value
Age		81.2 ± 9.2	81.5 ± 8.6	0.735
Number of drugs		13.3 ± 4.4	11.7 ± 4.3	0.061
Sex	Female	67 (79.8%)	71 (70.3%)	0.193
	Male	17 (20.2%)	30 (29.7%)	
Clinical complexity	Neither PCC nor MACA	33 (39.3%)	49 (48.5%)	0.453
	PCC	47 (56.0.1%)	48 (47.5%)	
	MACA	4 (4.8%)	4 (4.0%)	
GMA	1	0 (0.0%)	2 (2.0%)	0.291
	2	3 (3.6%)	9 (8.9%)	
	3	31 (36.9%)	41 (40.6%)	
	4	38 (45.2%)	36 (35.6%)	
	5	12 (14.3%)	13 (12.9%)	
ATC	A	15 (17.9%)	20 (19.8%)	0.191
	Others	1 (1.2%)	9 (9.0%)	
	C	10 (11.9%)	17 (16.8%)	
	M	2 (2.4%)	4 (4.0%)	
	N	56 (66.7%)	51 (50.5%)	

PCC: patients with chronic diseases and complex care; MACA: patients with advanced chronic disease and life-limiting prognosis; GMA: Adjusted Morbidity Group; ATC: Anatomical Therapeutic Chemical classification. Estimates are adjusted for age and number of active drugs; the outcome is full implementation of pharmacotherapeutic proposals.

**Table 2:** Primary and secondary 30-day health service utilization outcomes according to implementation status.

Endpoint/Outcome	No Implementation (n = 84)	Implementation (n = 101)
Primary endpoint		
30-day ED revisit (DRP-related)	9 (10.7%)	4 (4.0%)
Secondary endpoints		
30-day ED revisit (any cause)	19 (22.6%)	16 (15.8%)
30-day unplanned hospital readmission (DRP-related)	5 (6.0%)	4 (4.0%)
30-day unplanned hospital readmission (any cause)	12 (14.3%)	11 (10.9%)

DRP: drug-related problem; ED: emergency department.

## Data Availability

The original contributions presented in this study are included in the 391 article/Supplementary Materials. Further inquiries can be directed to the corresponding authors.

## Supplementary Materials

The following supporting information can be downloaded at: https://www.mdpi.com/article/10.3390/pharmacy14060134/s1, Table S1: Covariate balance before and after IPTW; Table S2: IPTW-adjusted association between implementation of pharmacotherapeutic proposals at hospital discharge and 30-day healthcare utilization outcomes.

## Abbreviations

The following abbreviations are used in this manuscript:

MC	Medication Code
DRP	Drug-related problem
ADE	Adverse drug event
ED	Emergency department
CST	Consorci Sanitari de Terrassa
IHO	Integrated Healthcare Organization
SIRE	Catalan regional electronic prescribing system
GMA	Adjusted Morbidity Group
PCC	Patients with chronic diseases and complex care
MACA	Patients with advanced chronic disease and life-limiting prognosis
ATC	Anatomical Therapeutic Chemical classification
PS	Propensity score
IPTW	Inverse probability of treatment weighting
PSM	Propensity score matching
OR	Odds ratio
CI	Confidence interval
ARR	Absolute risk reduction
NNT	Number needed to treat
