# Supplementary material for: Secondary Prevention Bundle for Drug-Related Problems in an Integrated Healthcare Setting: A Pragmatic Observational Implementation-Effectiveness Study
Source: Pharmacy (Basel). 2026 Sep 15;14(6):134. doi: 10.3390/pharmacy14060134 (PMC13610349; doi:10.3390/pharmacy14060134)
Supplement: Supplementary file 1 [file pharmacy-14-00134-s001.zip › pharmacy-4475426-supplementary.pdf]

Supplementary Table S1. Covariate balance before and after IPTW

Baseline Covariate	No Implementation (n = 84)	Implementation (n = 101)	Unadjusted SMD	Post-IPTW SMD	Balance Status (SMD < 0.10)
Age (years), mean (SD)	81.2 (9.2)	81.5 (8.6)	0.037	0.023	Achieved
Sex (% Male)	20.2%	29.7%	0.220	0.029	Achieved
GMA complexity tier, mean (SD)	3.7 (0.76)	3.49 (0.9)	0.261	0.017	Achieved
Number of active drugs, mean (SD)	13.2 (4.4)	11.7 (4.3)	0.365	0.045	Achieved
PCC/MACA status (%)	60.7%	51.5%	0.187	0.012	Achieved

SMD: standardized mean difference; IPTW: inverse probability of treatment weighting; GMA: Adjusted Morbidity Group; PCC: patients with chronic diseases and complex care; MACA: patients with advanced chronic disease and life-limiting prognosis; SD: standard deviation

Supplementary Table S2. IPTW-adjusted association between implementation of pharmacotherapeutic proposals at hospital discharge and 30-day healthcare utilization outcomes.

Endpoint/Outcome	Weighted risk (%) No Implementation	Weighted risk (%) Implementation	Risk difference (%)	IPTW OR (95% CI)	p-Value
**Primary endpoint**					
30-day ED revisit (DRP-related)	21.9	1.7	20.2	0.06 (0.01-0.3)	0.001
**Secondary endpoints**					
30-day ED revisit (any cause)	26.4	10.6	15.8	0.33 (0.15-0.73)	0.007
30-day unplanned hospital readmission (DRP-related)	11.5	1.7	9.9	0.13 (0.02-0.68)	0.016
30-day unplanned hospital readmission (any cause)	13.7	8.9	4.8	0.61 (0.24-1.55)	0.300

DRP: drug-related problem; ED: emergency department.
